# Breastfeeding in the first hour of life in Brazilian private hospitals participating in a quality-of-care improvement project

**DOI:** 10.1186/s12978-022-01538-z

**Published:** 2023-01-06

**Authors:** Rachael de Vasconcelos Alves, Maria Inês Couto de Oliveira, Rosa Maria Soares Madeira Domingues, Ana Paula Esteves Pereira, Maria do Carmo Leal

**Affiliations:** 1 Postgraduate Program in Public Health, Universidade Federal Fluminense, Rua Marquês de Paraná, no 303, Anexo, 4° Andar, Centro, Niterói, Rio de Janeiro, CEP: 24033-900 Brazil; 2 Department of Epidemiology and Biostatistics, Collective Health Institute, Universidade Federal Fluminense, Rua Marquês de Paraná, no 303, anexo, 3° Andar, Centro, Niterói, Rio de Janeiro, CEP: 24033-900 Brazil; 3 National Institute of Infectious Diseases Evandro Chagas, Fundação Oswaldo Cruz, Av. Brasil no 4365, Manguinhos, Rio de Janeiro, Rio de Janeiro CEP: 21040-360 Brazil; 4 National School of Public Health, Fundação Oswaldo Cruz, Rua Leopoldo Bulhões no 4480, Sala 814. Manguinhos, Rio de Janeiro, Rio de Janeiro CEP: 21041-210 Brazil; 5 National School of Public Health, Fundação Oswaldo Cruz, Rua Leopoldo Bulhões no 4480, Sala 809. Manguinhos, Rio de Janeiro, Rio de Janeiro CEP: 21041-210 Brazil

**Keywords:** Breastfeeding, Birth, Private hospitals, Cross-sectional studies

## Abstract

**Background:**

The Baby-Friendly Hospital Initiative’s Step 4 recommends: “support mothers to start breastfeeding as soon as possible after birth”, thus contributing to the reduction of neonatal mortality. The objective of this study is to estimate the prevalence of breastfeeding in the first hour of life in private maternity hospitals participating in the “Adequate Childbirth Project”, a quality-of-care improvement project, and to analyze determinants of this outcome.

**Methods:**

Secondary analysis of data collected by the cross-sectional evaluative “Healthy Birth Study”, conducted in 2017 in 12 maternity hospitals participating in the Adequate Childbirth Project, where 4800 mothers were interviewed, and hospital records were observed. Conditions that prevented breastfeeding at birth, such as maternal HIV-infection and newborns’ severe malformations, were excluded. Multiple logistic regression was performed according to a hierarchical theoretical model.

**Results:**

The prevalence of breastfeeding in the first hour of life was 58% (CI 95% 56.6–59.5%). Lower maternal education (aOR 0.643; CI 95% 0.528–0.782), lower economic status (aOR 0.687; CI 95% 0.504–0.935), cesarean section delivery (ORa 0.649; CI 95% 0.529–0.797), preterm birth (aOR 0.660; CI 95% 0.460–0.948) and non-rooming-in at birth (aOR 0.669; CI 95% 0.559–0.800) were negatively associated with the outcome. Receiving information during prenatal care about the importance of breastfeeding at birth (aOR 2.585; CI 95% 2.102–3.179), being target of the quality-of-care improvement project (aOR 1.273; CI 95% 1.065–1.522), skin-to-skin contact at birth (aOR 2.127; CI 95% 1.791–2.525) and female newborn (aOR 1.194; CI 95% 1.008–1.415) were factors positively associated with the outcome.

**Conclusions:**

The private maternities participating in the Healthy Birth Study showed a good prevalence of breastfeeding in the first hour of life, according to WHO parameters. Prenatal guidance on breastfeeding at birth, being target of the quality-of-care improvement project and skin-to-skin contact at birth contributed to breastfeeding in the first hour of life.

## Background

Breastfeeding reduces deaths of children under 5 years of age by 13% [1] and prevents child morbidity due to diarrhea and respiratory infections [2]. Even in high-income populations, its practice is important, as it reduces mortality from necrotizing enterocolitis and sudden infant death syndrome [3].

Breastfeeding at birth prevents the colonization of child’s gastrointestinal tract by gram-negative bacteria in the hospital environment. Colostrum contains immunological factors that protect the newborn and stimulate his active immune response [4]. A study carried out with data from 67 countries found an inverse correlation between breastfeeding in the first hour of life and neonatal mortality [5]. A survey conducted in Ghana with 10,947 children showed a 22% reduction in neonatal mortality associated with breastfeeding in the first hour of life, compared to those who started breastfeeding after 24 h [6]. A study of 37,350 children, carried out from the II Human Development Survey of India, showed an almost 3 times higher risk of mortality in non-breastfed children in the first hour of life [7].

The Baby-Friendly Hospital Initiative motivates facilities providing maternity and newborn services worldwide to implement the Ten Steps to Successful Breastfeeding. Step 4 recommends: “Facilitate immediate and uninterrupted skin-to-skin contact and support mothers to initiate breastfeeding as soon as possible after birth”. The skin-to-skin contact should remain after birth for at least one hour, mothers being encouraged to identify whether the baby shows signs of wanting to be breastfed, and help should be offered, if necessary [8]. This was the theme of the World Breastfeeding Week in 2007: “Breastfeeding: the 1st Hour – Save ONE million babies!” [9]. In Brazil, the National Demography and Health Survey, carried out in 2006, found that 43% of children started breastfeeding in the first hour of life [10], while in 2008, in the Brazilian capitals, this proportion was 67.7% [11]. In the “Birth in Brazil” survey, conducted between 2011 and 2012, 56% of children born in hospitals with more than 500 births/year (corresponding to 78.6% of hospital births) were breastfed in the first hour of life [12].

Systematic reviews [13, 14] point to cesarean section delivery as the most important risk factor for not breastfeeding in the first hour of life. This is worrying, since in 2008, Brazil contributed with 15% of the total unnecessary cesarean sections that occurred in the world [15]. Birth in a private maternity also proved to be a risk factor for delayed initiation of breastfeeding, while delivery in a Baby-Friendly Hospital was a protective factor [13].

The high contribution of the Brazilian private sector to the performance of cesarean sections and consequent neonatal outcomes stimulated the creation of the “Adequate Childbirth Project”, aiming at prenatal and childbirth care improvement and reduction of the number of cesarean sections and hospitalizations in neonatal ICU [16]. The project was structured into four components: governance, women's empowerment, reorganization of care and monitoring [17]. The Healthy Birth Study assessed the degree of implementation and the effects of this project.

In Brazil, 30% of mothers give birth in the private sector, but private maternity hospitals breastfeeding practices, specially at birth, as well the factors associated with these practices, are seldom studied. The present study innovates investigating the prevalence of breastfeeding in the first hour of life in private maternity hospitals participating in a quality-of-care improvement project and analyzed the determinants of this outcome.

## Methods

This study is a secondary analysis of data collected by the Healthy Birth Study, a cross-sectional evaluative investigation carried out in 2017, 18 months after the beginning of the implementation of the Adequate Childbirth Project (ACP).

The Healthy Birth Study selected a convenience sample of 12 hospitals from the 23 private hospitals that joined the project to improve prenatal and childbirth care. For the selection of these hospitals, three criteria were considered: location of the hospital by geographic macro-region (at least one hospital from the northeast, southeast and south regions), type of hospital (owned or not by a health insurance company) and performance of the hospital in the prenatal and childbirth care improvement project (hospitals that reported good and bad results in achieving the ACP goals, according to administrative data provided by the project coordination board, were selected) [18]. Among the hospitals participating in the study, one was situated in the northeast region, nine in the southeast region and two in the south of Brazil.

The Healthy Birth Study’s sample was calculated to detect a 10% reduction in the proportion of cesarean sections, using a 50% cesarean rate as a reference, with 80% power and a 5% significance level. Overall, the sample size of 4800 women (12 hospitals times 400 women) had an accuracy of 80% in detecting a 2.5% reduction in the prevalence of cesarean sections. Losses and refusals accounted for about 5% of women and were replenished to complete 400 women in each hospital.

Electronic forms using the REDCap [19] application were developed. A pilot study was carried out in one of the maternity hospitals participating in the prenatal and childbirth care improvement project not included in this evaluative research. The questionnaires were tested and the logistical aspects of the fieldwork were refined.

Trained interviewers, external from the hospitals, conducted data collection, addressing all women admitted to the hospital who met the eligibility criteria to participate in the study, until 400 participants were included in each hospital. Women who did not speak Portuguese, with hearing loss, whose delivery occurred outside the hospital and who were hospitalized for judicial termination of pregnancy were not eligible for the study. The women were interviewed face to face at least 6 h after vaginal delivery and 12 h after cesarean section, after reading and signing an informed consent form.

Three instruments were used: 1. questionnaire applied to the puerperal woman with questions related to maternal characteristics, pregnancy and prenatal care, childbirth care, newborn and infant feeding; 2. form for extracting data from the medical record of the puerperal woman and the newborn about the type of pregnancy, type of delivery, condition of the newborn and feeding of the newborn during hospitalization; 3. form for extracting data from the prenatal card [18].

In prolonged hospitalizations, data were collected from medical records until the 28th day of infant hospitalization and on the woman's 42nd day of hospitalization. In the case of hospital transfer, data were obtained from the medical records of the hospital from which the puerperal woman and/or the newborn were discharged. Data collection was conducted from March to August 2017. Due to the variation in the size of hospitals, the time required for data collection ranged from 1 to 4 months, depending on the total number of births per month in each participating hospital [18].

The present study used as inclusion criteria living newborns with gestational age ≥ 34 weeks (stillborns: n = 19). Newborns or women who met one or more of the following criteria were excluded: newborns with malformations that could disturb or prevent breastfeeding (malformations: n = 47), sons of HIV-infected mothers (n = 6), postpartum women with severe maternal morbidity or transferred to the Intensive Care Unit (ICU) due to complications during delivery (n = 244) and babies transferred to the ICU at birth (n = 527).

The excluded malformations were severe congenital heart disease, Down syndrome, craniofrontonasal syndrome, hydrocephalus, cleft lip or soft palate cleft, pulmonary cystic adenomatosis, corpus callosum dysgenesis, gastroschisis, diaphragmatic hernia, vermin hipoplasia, omphalocele and Moebius and Robin sequence.

A total of 785 women were excluded, and data from 4,093 binomials were analyzed. Post-hoc calculations showed that, considering a prevalence of exclusive breastfeeding of 60% and a significance level of 5%, the sample after the exclusions used in the present analysis had 90% power to detect 5% differences in the proportion of this outcome.

**Fig. 1:**
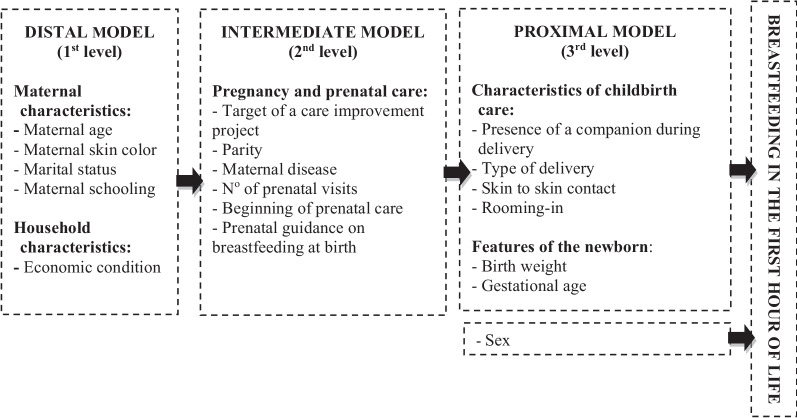
Theoretical hierarchical model of factors associated with breastfeeding in the first hour of life

The outcome was breastfeeding in the first hour of life (yes/no), obtained from two questions to the puerperal woman: "After birth, did you breastfeed in the delivery room?" and "How long, more or less, did it take you to breastfeed for the first time?" Breastfeeding in the first hour of life was considered when the answer to the first question was “yes” and/or the second covered up to 1 h. A hierarchical theoretical model was adopted, with maternal and household characteristics as distal exposure variables; pregnancy and prenatal care characteristics as intermediary exposure variables and hospital, delivery, and newborn characteristics as proximal exposure variables (Fig. 1).

The selected maternal characteristics were age (< 20; 20–34; ≥ 35), self-reported skin color (white; black; brown/yellow/indigenous), having a partner (single/separated/widowed; married/living with a partner), education (up to 14 years; ≥ 15 years). Household characteristics were analyzed based on economic conditions (class A—above average; B—average; C + D + E—below average) [20]. Characteristics of pregnancy and prenatal care were parity (primiparous; multiparous), being target of the Adequate Childbirth Project (yes; no), maternal disease—hypertension, diabetes, or other chronic diseases (yes; no), number of prenatal consultations (≤ 7; 8 or more), beginning of prenatal care (up to 12 weeks; ≥ 13 weeks), guidance on breastfeeding during prenatal care (yes; no). The characteristics of childbirth care selected were presence of a companion during delivery (yes; no/did not want), type of delivery (vaginal; cesarean section), skin-to-skin contact at birth (yes; no) and rooming-in (measured by the question: “did the baby go to the room with the mother?”: yes; no). Features of the newborn selected were birth weight (< 2500 g; ≥ 2500 g), gestational age (34–36 weeks; ≥ 37 weeks) and sex (male; female).

Different groups of women were target of the Adequate Childbirth Project, as defined by the hospital manager, such as: 1. primiparous women; 2. pregnant women belonging to Robson Groups 1 to 4 (nulliparous or multiparous without previous cesarean section, single fetus, term, cephalic, according to WHO [20]) because these women had greater chances of vaginal delivery; 3. women served by the on-call team; 4. pregnant women without previous uterine scarring whose delivery was performed by the on-call team [18].

All statistical analyses were performed using SPSS software version 17 (https://www.Ibm.com/), using data weighting and incorporating the design effect, considering the complex sampling process [18]. Considering the sample weight, the final number analyzed was 4060 binomials.

Initially, univariate analysis was conducted to identify the distribution of exposure variables and the outcome under investigation. Then, bivariate analysis was applied between each exposure variable and the outcome, using Pearson's chi-square test and crude odds ratios (OR) were obtained with their respective 95% confidence intervals (95% CI). The variables that obtained p ≤ 0.20 in the bivariate analysis were included in the statistical modeling. Finally, multiple logistic regression was conducted, following the hierarchical conceptual theoretical model applied, according to the temporal proximity of each variable with the outcome [21].

## Results

The prevalence of breastfeeding in the first hour of life was 58.0% (CI 56.6–59.5%). There was a wide variation between hospitals, five maternities with prevalence rates above 80% and three less than 30%. There was also discrepancy within the regions, as both the highest (93.0%) and the lowest prevalence (6.3%) were found in the Southeast Region.

Most of the women had ages between 20 and 34 years, were white, lived with a partner, had a high level of education and belonged to economic class B. The distal factors associated with the outcome (p < 0.20) in the bivariate analysis were age, skin color, marital status, education level and socioeconomic class (Table 1).

**Table 1:** Prevalence of breastfeeding in the first hour of life and crude odds ratio according to sociodemographic characteristics

Variable	n	%	BF (%)	OR	95% CI	p-value
Maternal age						
< 20 years	63	1.5	51.5	0.819	0.497–1.350	0.434
20–34 years	2643	64.0	56.4	1		
35 years or more	1421	34.4	61.2	1.068	1.053–1.395	0.003
Skin color*						
Black	196	4.8	45.6	0.528	0.397–0.701	< 0.001
Brown/yellow/indigenous	1116	27.0	49.8	0.650	0.567–0.746	< 0.001
White	2814	68.2	62.1	1		
Marital status						
Single/separated/widowed	262	6.4	46.5	0.656	0.514–0.838	< 0.001
Living with a partner	3848	93.2	58.9	1		
Maternal schooling						
Up to 14 years	1699	42.0	45.6	0.395	0.348–0.450	< 0.001
≥ 15 years	2345	58.0	67.2	1		
Economic condition						
Class C. D or E	663	16.3	42.7	0.276	0.226–0.338	< 0.001
Class B	2231	55.0	54.4	0.450	0.383–0.528	< 0.001
Class A	1166	28.7	73.7	1		
Total	4060	100	58.0			

Northeast, Southeast and South Regions, Brazil, 2017

*BF* breastfeeding in the first hour of life

*Classification of skin color used by the Brazilian Demographic Census

Concerning the intermediate characteristics, more than half of the mothers were target of the Adequate Childbirth Project, almost 60% of the women were primiparous, just over a tenth had some pathology (hypertension, diabetes, or other chronic disease), the majority started prenatal care in the first trimester and was accompanied by 8 or more prenatal visits. About three quarters of women received prenatal guidance on breastfeeding at birth. Being target of the Adequate Childbirth Project, maternal pathology, number of prenatal visits, beginning of prenatal care and receiving prenatal guidance on breastfeeding in the first hour of life were associated with the outcome in the bivariate analysis (Table 2).

**Table 2:** Prevalence of breastfeeding in the first hour of life and crude odds ratio according to characteristics of pregnancy and prenatal care

Variable	n	%	BF (%)	OR	95% CI	p-value
Target of a care improvement project						
Yes	2214	54.5	61.9	1.558	1.373–1.767	< 0.001
No	1846	45.5	53.4	1		
Parity						
Primiparous	2398	59.1	57.8	1		
Multiparous	1658	40.9	58.4	0.976	0.859–1.108	0.709
Maternal disease						
Yes	469	11.5	48.8	0.666	0.551–0.805	< 0.001
No	3592	88.5	59.2	1		
Number of prenatal visits						
0 to 7 visits	345	8.5	46.1	0.540	0.434–0.673	< 0.001
8 or more visits	3714	91.5	59.1	1		
Beginning of prenatal care						
1st gestational trimester	3838	94.8	58.8	1		
2nd or 3rd trimester	210	5.2	44.3	0.508	0.385–0.670	< 0.001
Prenatal guidance on breastfeeding at birth						
Yes	3158	77.8	64.9	3.420	2.932–3.989	< 0.001
No	902	22.2	33.9	1		

Northeast, Southeast and South Regions, Brazil, 2017

*BF* breastfeeding in the first hour of life

Most women had a companion during delivery and more than three quarters underwent cesarean section. Most newborns were born at term, the sex ratio was similar, about 3% had low birth weight and almost 60% had skin-to-skin contact with the mother at birth. The majority (64.2%) of the newborns did not follow to rooming-in directly after birth, staying for a while in a nursery/heated crib/incubator. In bivariate analysis, among proximal characteristics, type of delivery, sex of the baby, preterm birth, low birth weight, skin-to-skin contact with the mother and rooming-in were associated with breastfeeding in the first hour of life (Table 3).

**Table 3:** Prevalence of breastfeeding in the first hour of life and crude odds ratio according to hospital characteristics, delivery, and newborn care

Variable	n	%	BF (%)	OR	95% CI	p-value
Presence of a companion during delivery						
Yes	3982	98.7	58.2	1,39	0.866–2.245	0.171
No/did not want	63	1.3	47.6	1		
Type of delivery						
Cesarean section	3120	76.8	52.5	0.367	0.312–0.432	< 0.001
Vaginal	940	23.2	76.3	1		
Sex						
Female	2818	49.7	59.6	1.137	1.003–1.288	0.045
Male	2041	50.3	56.5	1		
Low birth weight						
Yes (< 2500 g)	117	2.9	50.4	0.724	0.498–1.052	0.090
No (2500 g or more)	3920	97.1	58.3	1		
Preterm birth						
Yes (34–36 weeks)	213	5.2	46.9	0.682	0.515–0.903	0.007
No (≥ 37 weeks)	3847	94.8	58.6	1		
Skin to skin contact						
Yes	1817	59.7	78.9	2.634	2.239–3.098	< 0.001
No	1229	40.3	55.9	1		
Rooming-in directly after birth						
Yes	1447	35.8	69.0	1		
No	2598	64.2	52.0	0.490	0.427–0.562	< 0.001

Northeast, Southeast and South Regions, Brazil. 2017

*BF* breastfeeding in the first hour of life

In the multivariate analysis, women with lower education, economic status below average, who underwent cesarean section, whose newborn had gestational age between 34 and 36 weeks and those that did not follow directly to rooming-in had lower rates of breastfeeding in the first hour of life. Being target of the Adequate Childbirth Project, receiving prenatal guidance about breastfeeding at birth, female newborn and skin-to-skin contact at birth increased the chances of breastfeeding in the first hour of life (Table 4).

**Table 4:** Adjusted odds ratio of breastfeeding in the first hour of life

	aOR	95% CI	p-value
Distal variables			
Maternal schooling			
Up to 14 years	0.643	0.528–0.782	< 0.001
≥ 15 years	1		
Economic condition			
Class C. D or E	0.687	0.504–0.935	0.017
Class B	0.853	0.693–1.051	0.136
Class A	1		
INTERMEDIATE VARIABLES			
Target of a care improvement project			
Yes	1.273	1.065–1.522	0.008
No	1		
Prenatal guidance on breastfeeding at birth			
Yes	2.585	2.102–3.179	< 0.001
No	1		
Proximal variables			
Type of delivery			
Cesarean section	0.649	0.529–0.797	< 0.001
Vaginal	1		
Sex			
Female	1.194	1.008–1.415	0.049
Male	1		
Preterm birth			
Yes (34–36 weeks)	0.660	0.460–0.948	0.024
No (≥ 37 weeks)	1		
Skin to skin contact			
Yes	2.127	1.791–2.525	< 0.001
No	1		
Rooming-in directly after birth			
Yes	1		
No	0.669	0.559–0.800	< 0.001

Northeast, Southeast and South Regions, Brazil, 2017

*BF* breastfeeding in the first hour of life

## Discussion

In private maternity hospitals participating in the Healthy Birth Study 58% of newborns were breastfed in the first hour of life, a percentage classified as “good” (50–89%), according to WHO parameters [22]. This prevalence stands between that found by the National Demography and Health Survey in 2006 (42.9%) [11] and by the survey carried out in 2008 in the capitals and the Federal District (67.7%) [10]. It is similar to the prevalence of 57.8% verified in the WHO Global Maternal and Perinatal Health Survey covering 24 countries in Africa, Asia and Latin America [23] and well above that found by other investigations in private maternity hospitals. In the study “Birth in Brazil”, carried out between 2011 and 2012, 25.3% of newborns in private maternity hospitals were breastfed in the first hour of life [24], while in 1999/2001, in 25 private hospitals in the city of Rio de Janeiro, this prevalence was only 1.6% [25].

Despite the unfavorable scenario of private maternity hospitals, where the practice of elective cesarean section is very high [26], the quality-of-care improvement project increased the prevalence of breastfeeding by almost 30% in the first hour of life in the target group, reflecting advances in the reorganization of prenatal and childbirth care. The prevalence of breastfeeding at birth in private maternity hospitals before the Adequate Childbirth Project was much lower, around 26% [24]. Meantime not all hospitals surveyed seemed to respond in the same way. A quarter of hospitals had a low prevalence (< 30%), according to WHO parameters [22], while in half of them more than 60% of babies suckled in the first hour of life.

Other factors were also associated with the outcome. The prevalence of breastfeeding in the first hour of life in women with less than 15 years of schooling was about 35% lower than those with higher schooling. Probably, higher educational levels allow greater access to information on the benefits of breastfeeding at birth [27, 28]. In a systematic review that brought together studies from Asia, Africa and South America, low maternal education was also a risk factor for not breastfeeding in the first hour of life [13]. In the same direction, a dose–response effect was found between economic class and the prevalence of breastfeeding at birth, women from classes C, D and E having breastfed about 30% less than women from class A and women from class B 15% less. The more unfavorable the socioeconomic stratum, the lower this practice was shown [13].

Receiving prenatal guidance on breastfeeding in the first hour of life more than doubled the prevalence of the outcome. Guidance on breastfeeding during prenatal care proved to be an important factor to improve breastfeeding at birth in several studies [12, 13, 28–31], because during pregnancy women need guidance and support to breastfeed [13, 29].

Cesarean section was one of the factors most associated with the outcome, in the present study and in other settings, decreasing the prevalence of breastfeeding in the first hour of life by almost 40%. Cesarean section appears as a risk factor [13] related to postpartum pain [14, 32, 33], less skin-to-skin contact with the mother [14, 34], maternal anesthesia [25, 28, 29, 35, 36], difficulty in holding the child soon after birth [29] or postpartum surgical procedures, which delay and often interrupt mother-baby contact [12, 25, 37]. Mothers with vaginal delivery may have a more active participation in the breastfeeding process at birth, facilitating skin-to-skin contact, recognizing signs that the newborn is able to be breastfed and releasing oxytocin [24, 34, 38]. The Adequate Childbirth Project was effective in reducing the percentage of cesarean sections in the maternity hospitals studied, with an overall reduction of 10% in the event compared to the same maternities at the beginning of the quality-of-care improvement project [39], contributing to the increase in the prevalence of breastfeeding in the first hour of life in that context.

Female babies were almost 20% more likely to be breastfed in the first hour of life when compared to male babies. Boccolini et al [40] found a similar association in a Brazilian study, that may be attributed to a higher probability of adverse events in pregnancy among male children, or to cultural beliefs, as female babies being expected to suck the breast more gently than male ones.

Late preterm newborns (gestational age 34–36 weeks) had 35% less chance of being breastfed at birth when compared to those born at term. Vieira et al [29] argue that preterm newborns are less likely to be breastfed in the first hour of life, as they are sleepier, do not coordinate well suction-breath-swallowing and have less suction reflex. Even early term newborns seem to have some difficulty in establishing exclusive breastfeeding in relation to those born from 38 weeks of gestational age, as they have a greater chance of adverse events, such as respiratory diseases and longer hospital stay [41]. Newborns of spontaneous labor tend to be born at a higher gestational age, since elective caesarean sections may be contributing to the reduction of gestational age [42].

Skin-to-skin contact between mother and child at birth doubled the prevalence of breastfeeding in the first hour of life. In Rio de Janeiro, a similar result was observed, hospital routines being one of the reasons that prevent this early contact with the mother [25]. Lau [33] in a study in Singapore found that skin-to-skin contact in the first 30 min of life had a positive association with breastfeeding in the first hour of life, both in cesarean sections and in vaginal deliveries. Skin-to-skin contact between mother and child is also important for the maintenance of the baby's body temperature, for cardiorespiratory stability and to bond mother and child, facilitating the establishment of early initiation of breastfeeding [33].

More than half of the newborns did not remain rooming-in since birth, not because of the severity of the babies, since only 3.2% of them went to the semi-intensive neonatal unit, about 80.7% being allocated in a nursery/heated crib/incubator after birth. In a Brazilian study, the prevalence of rooming-in in private maternity hospitals was also low (51.3%) [24]. Rooming-in has been an international recommendation since the 1950s [43], adopted in Brazil as a law since 1993 [44]. Among the benefits of rooming-in is greater contact between mother and baby, which enhances autonomy to understand and to take care of the child, in addition to more opportunities of interaction with the health team [40, 45].

The present study has limitations. Cross-sectional studies not always allow the establishment of a causal relationship between exposure variables and the outcome, however, most of the variables studied have a clear temporality relationship with breastfeeding at birth. Intentionally, a convenience sample of private maternity hospitals was adopted, located in three Brazilian regions, not covering the North and Midwest regions, which impairs their representativeness in relation to the private network.

## Conclusions

We conclude that the private maternity hospitals participating in the Healthy Birth Study had a good prevalence of breastfeeding in the first hour of life, according to WHO parameters [22]. Prenatal guidance on breastfeeding at birth, being target of a project for improving the quality of care and immediate skin-to-skin contact contributed to the outcome, showing the importance of implementing these actions for breastfeeding in the first hour of life. Other factors related to women’s conditions and to delivery were associated with the outcome in the multivariate model, indicating that they should also be object of care to improve this practice.

We recommend a greater investment in expanding the practice of breastfeeding in the first hour of life, vital for child's health and wellbeing. Improving quality-of-care models should be encouraged, so that the private sector can be supported to reorganize prenatal and childbirth care in a humanization view. Good hospital care practices for childbirth are necessary, with the creation of mechanisms that stimulate vaginal delivery, skin-to-skin contact and immediate rooming-in, to favor breastfeeding at birth.

## Data Availability

The datasets used and analyzed during the current study are available from the corresponding author on reasonable request.
